# Workplace Violence Against Doctors in Bangladesh: A Content Analysis

**DOI:** 10.3389/fpsyg.2021.787221

**Published:** 2021-12-03

**Authors:** Shirmin Bintay Kader, Md. Marufur Rahman, Md. Khaledul Hasan, Md. Mohibur Hossain, Jobaida Saba, Sophia Kaufman, Enryka Christopher, Kamrun Nahar Koly

**Affiliations:** ^1^Health Systems and Population Studies Division, International Center for Diarrheal Disease Research, Bangladesh (icddr,b), Dhaka, Bangladesh; ^2^Center for Medical Biotechnology, Management Information System, Directorate General of Health Services, Dhaka, Bangladesh; ^3^Department of Oncology and Metabolism, The Medical School, University of Sheffield, Sheffield, United Kingdom; ^4^Department of Oncology, Bangabandhu Sheikh Mujib Medical University (BSMMU), Dhaka, Bangladesh; ^5^Department of Public Health and Informatics, Jahangirnagar University, Dhaka, Bangladesh; ^6^Department of Social Anthropology, Harvard University, Cambridge, MA, United States; ^7^Departments of History and Philosophy of Science, Sociology and Social Anthropology, Sidney Sussex College, University of Cambridge, Cambridge, United Kingdom; ^8^Department of Psychiatry, Boston Children’s Hospital and Harvard Medical School, Boston, MA, United States

**Keywords:** doctors, workplace violence, patient behavior, content analysis, Bangladesh

## Abstract

Workplace violence in healthcare settings is a common global problem, including in Bangladesh. Despite the known presence of workplace violence in healthcare environments of developing countries, there is limited understanding of factors that lead to hospital violence in Bangladesh. This study aims to explore factors that influence incidents of violence against healthcare professionals in Bangladesh, as reported by doctors *via* social media forum. Content analysis was conducted on 157 reported incidents documented on “Platform,” the online social media most used by medical students and doctors in Bangladesh. Posts by doctors detailing experiences of physical or verbal violence at their workplace between July 2012 and December 2017 were included in this study. The majority of reported incidents were reported by male doctors (86%) and from government hospitals (63.7%). Findings showed that primary healthcare centers experienced more violence than secondary and tertiary facilities. This may largely be due to insufficient human and other resources in primary care settings to meet patient demand and expectations. Most of the events happened at night (61%), and as a result, entry-level doctors such as emergency duty doctors and intern doctors were commonly affected. Six themes were identified as vital factors in workplace violence against doctors: patients’ perspectives, delayed treatment, power practice, death declarations, extreme violence, and care-seeking behaviors. Most incidents fell under the categories of delayed treatment and power practice at 26.8 and 26.1%, respectively. This study identified possible factors for reported violence in hospital settings. To address and reduce these incidents, hospital administrators should be aware of risk factors for violent behavior and design appropriate measures to prevent workplace violence. Further qualitative and quantitative research is needed to appropriately address the consequences of violence on healthcare workers and implement measures to mitigate these events.

## Introduction

Workplace violence (WPV) in healthcare settings is becoming a global problem that traverses geographic borders and is indiscriminate of levels of care (Fernandes et al., 1999; Kowalenko et al., 2005; Behnam et al., 2011; Wu et al., 2012; Arnetz et al., 2015; Kumar et al., 2016; Abdellah and Salama, 2017). The National Institute for Occupational Safety and Health (NIOSH) has addressed WPV as any violent act (either physical or verbal) that is directed toward a person at work or while on duty (NIOSH, 2002).

WPV is a global phenomenon that extends to both developed and developing countries (Wiskow, 2003). According to a national survey conducted in the United States (US), 78% of emergency department doctors confirmed that they were the target of workplace violence. Among reported incidents, 75% were categorized as verbal assault, 21% were physical assault, 5% were confrontations outside of the hospital facility, and 2% were cases of abuse or harassment (Behnam et al., 2011). Another US-based study revealed that nearly 75% of interviewed doctors had faced verbal threats at least once in the past twelve months (Kowalenko et al., 2005). Additionally, a study conducted by the British Medical Association (BMA) in the United Kingdom (UK) found that one-third of the doctors interviewed had faced WPV in the past year (Pitcher, 2008). The Indian Medical Association also reported that up to 75% of doctors faced some sort of violence in their workplace (Reddy et al., 2019; Dora et al., 2020). Besides, any suffering related to the workplace can demotivate the healthcare providers, affecting the quality of care (Sánchez-Hernández et al., 2020).

The Bureau of Labor Statistics (BLS) reported that in the United States, health workers faced WPV at a rate four times higher than non-health workers (BLS, 2012). A nationwide survey identified verbal abuse as the most common act of violence committed against doctors or other hospital staff in the United States, followed by physical assaults (Kowalenko et al., 2005; Behnam et al., 2011). Gerberich et al. (2004) suggest that in the United States, only 15% of violent events that occur within the health sector are reported (Gerberich et al., 2004). The nature of WPV in developing countries is similar; most doctors have experienced verbal and physical abuse from patients’ relatives (Kumar et al., 2016; Abdellah and Salama, 2017). More than 75% of doctors working in a tertiary hospital in New Delhi, India, reported that experiencing a violent incident impacted their mental state and disrupted their everyday lives (Kumar et al., 2016). A study conducted in Pakistan found that 73.8% of healthcare workers in public sector hospital settings were exposed to aggression and violence (Imran et al., 2013). Not only do hospitals and administrators often ignore violence taking place in their organizations, but doctors also avoid sharing their experiences, resulting in fewer reports filed, which further administrative ignorance (Arnetz et al., 2015).

In lower-middle-income countries like Bangladesh, doctors, nurses, and other healthcare workers often encounter multiple experiences of physical or verbal abuse in their workplace. According to the WHO, the doctor-patient and nurse–patient ratios in Bangladesh are very low, with only 3.05 doctors and 1.07 nurses per population of 10,000 (WHO, 2021). Healthcare staffs are subsequently overburdened, with the average consultation time being less than a minute for outpatients. Access to essential services is also limited (Irving et al., 2017). More than 24% of patients experience a treatment delay of up to 4 h in hospitals (Ali et al., 2013). Due to insufficient funds, 34% of the ideal number of staff posts in the health sector are vacant (Fahim et al., 2019). Frustrations with the healthcare system are commonplace due to low financial and human resources, which doctors are often blamed for. There have been many cases where a patient’s family or friends act violently toward the doctor when a patient dies (Naik, 2013; Kumar and Roy, 2019).

In 2020, a doctor in Bangladesh was murdered by a patient’s family member. The family member accused the doctor of malpractice when the patient died under the doctor’s care (Prothomalo, 2020). Although a few researchers have started to address the issue of WPV in non-western contexts, no studies have yet been conducted to investigate the nature, causes, or impacts of WPV within Bangladesh (Khan et al., 2010; Hossain et al., 2018). The current study aimed to compile incidents of WPV shared by Bangladeshi doctors in an existing large social media forum for doctors, called “Platform.” Findings from this study can help generate information on catalysts of WPV and guide policies and interventions to prevent such events. Ensuring a safer workplace for frontline health workers in Bangladesh will contribute to a safer society.

## Materials and Methods

### Data Collection

For data collection, we scrutinized the posts on an online forum called Platform, a social media for registered doctors of Bangladesh to share medical science and public health content. Imran et al. elaborately described the organizational structure of Platform; it is a non-governmental, non-profit forum that is open to all medical doctors and medical students of Bangladesh (Hasan et al., 2018). The social experiences that doctors have while working in the field of medical science are shared across this forum. We performed a retrospective scanning from January 2018 to March 2018. During the scanning in the Platform social media forum, initially, we only considered the reported news/posts regarding any kind of violence, including physical assault, verbal abuse, and hospital vandalism at any healthcare setting of Bangladesh. Later, one of our co-authors separated only those reports, which had the complete elaboration of the reported events. We also have cross-checked the incidence with the published news on daily newspaper portals and pulled additional information if needed to ensure the validity of the data.

We retrieved and reviewed a total of 642 Platform posts reported from July 2012 to December 2017. Among those, 273 posts were related to unpleasant incidents that occurred at healthcare facilities. From these unpleasant incidents, 162 described a WPV incident in detail, written by the doctors who experienced them. Duplicated posts were identified and excluded, leaving 157 posts for the final dataset. All posts were translated to English, and a data analyst removed all personal information to maintain participants’ confidentiality.

### Data Analysis

This study used conventional content analysis (Hsieh and Shannon, 2005) on the selected 157 posts that reported a violent event. Posts were reviewed repeatedly for familiarization of response patterns (Hsieh and Shannon, 2005). A data-driven inductive approach supported thematic content analysis (Boyatzis, 1998). Two research staff conducted all coding using MS excel and frequently met to review and discuss coding schema to ensure a robust quality of data interpretation. After coding was completed, a third researcher, who was blind to previous coding and sub-coding, reassessed the analysis.

## Results

**Figure 1:**
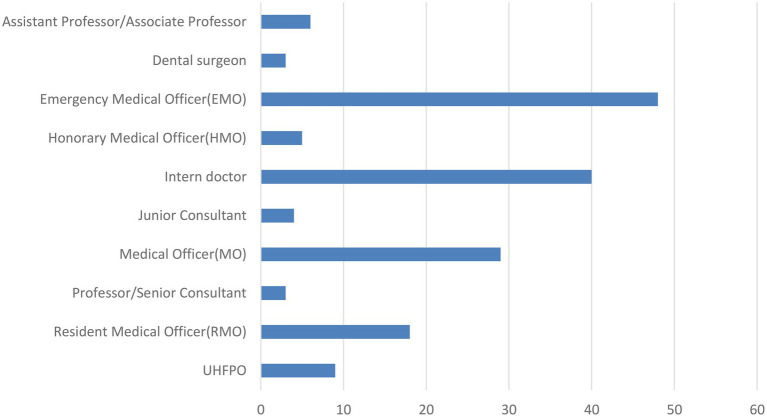
Different level of injured doctors during the violence.

**Figure 2:**
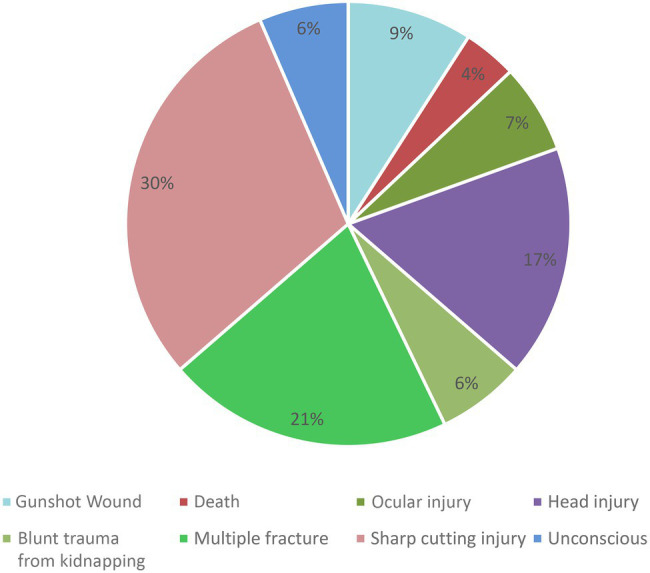
Types of injury due to workplace violence (*N*=77).

**Figure 3:**
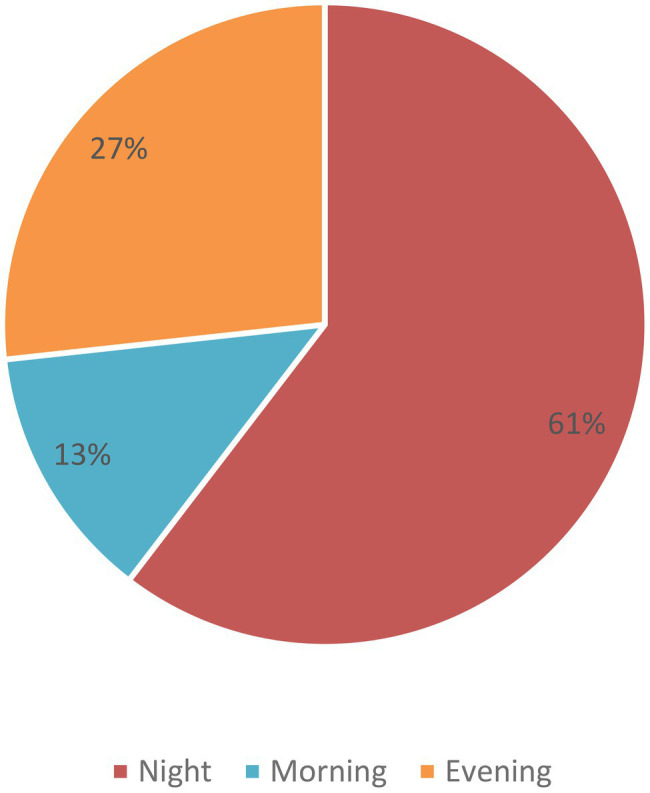
Distribution of the workplace violence according to the duty roaster of hospitals.

Among the 157 total incidents, the male and female doctor’s ratio was 86% (135) and 14% (22), respectively (Table 1). In these 157 incidents, we found that a total of 165 doctors got injured from different levels of healthcare facility centers. Among these doctors, we observed that the entry-level doctors like intern doctors and emergency medical officers were injured mostly due to WPV (Figure 1). We found that sharp cutting injury, multiple fractures, and head injury occurred mostly. We also found that even a few fatal injuries like “died on duty” were reported in three cases (Figure 2). Most of the reported incidents occurred at night (61%), and the rest of the reported incidents occurred in the evening (27%) and morning (13%; Figure 3).

**Table 1:** Sex distribution of the doctors reported the events at different level of hospitals in Bangladesh.

Sex	Primary level (%)	Secondary level (%)	Tertiary level (%)	Total (%)
Male	77 (49)	20 (13)	38 (24)	135 (86)
Female	10 (6)	8(5)	4 (3)	22 (14)
**Total**	**87 (55)**	**28 (18)**	**42 (27)**	**157 (100)**

Six major themes were identified (patients’ perspective, delayed treatment, power practice, death declarations, extreme violence, and care-seeking behavior). More than half of the events occurred within two themes: delayed treatment (27%) and power practice (26%). The least number of events were identified under extreme violence, which included murder and sexual assault. Private healthcare facilities made up 36.7% of the locations of reported events, while the rest were from government-funded health systems. In terms of facility levels, more than half of reported incidents took place in primary healthcare facilities, 18% were reported from secondary healthcare facilities, and 27% of the reported events occurred in tertiary healthcare facilities (Table 2).

**Table 2:** Distribution of the violent incidents at different level of hospitals in Bangladesh.

Theme	No. of incidents (%)	Primary level (%)	Secondary level (%)	Tertiary level (%)
Patients’ perspectives	19 (12)	13 (68)	3 (16)	3 (16)
Delayed treatment	42 (27)	24 (57)	11 (26)	7 (17)
Power practice	41(26)	24 (59)	9 (22)	8 (20)
Death declaration	28 (18)	11 (39)	2 (7)	15 (54)
Extreme violence	8 (5)	3 (38)	0 (0)	5 (63)
Care-seeking behavior	19 (12)	12 (63)	3 (16)	4 (21)
**Total**	**157 (100)**	**87 (55)**	**28 (18)**	**42 (27)**

### Patients’ Perspectives

We explored reasons speculated by doctors for contributing to patients’ or patient parties’ aggressive behavior against healthcare providers. About 12% of the reviewed data included posts that mention patient dissatisfaction with their proposed treatment plan as the likely catalyst for aggressive behavior. The majority of the posts posited that patient death following intravenous or intramuscular drug injection triggered the patients’ family or friends to act violent, as they misattributed the death to the administration of drugs by the doctor.

In the words of one doctor from a primary healthcare center:

*“When the patient came to me, his condition was so critical. I could not manage that patient at our facility. So, I suggested they (relatives of the patient) take him to a district-level hospital immediately, but they refused and forced me to manage the patient in that condition. I felt helpless but attempted to stabilize the patient with an injection. Immediately after, his condition improved slightly, so I requested that they take him to a secondary-level hospital, but they still refused. Unfortunately, the patient died a few moments later. They (relatives of the patient) became very violent and broke our hospital and the residence of our Upazila Health and Family Planning Officer (UHandFPO). They chased our staff and a few of them were injured”* (Case 23, Male doctor).

In some of these patient death cases, the patients’ party specifically accuse the doctor of medical malpractice. One incident report from a primary healthcare setting read:

*“A mother who underwent a caesarean section died the next morning after her surgery. The gynaecologist was accused of prescribing the wrong medication and injection. The relatives of the patient claimed that the mother died after getting the dose. Only the baby survived”* (Case 11, Male doctor).

The general public’s limited understanding of the risks medical procedures carries often leaves surviving family or friends with little clarity about how such tragedies could occur to their loved ones. Grief counselling is rarely provided, patient advocates are nonexistent, and even legitimate concerns about malpractice are often overlooked with healthcare resources already strained. It is within this context that patient deaths can create confusion, which lead to subsequent violent acts.

### Delayed Treatment

More than a quarter (26.8%) of the 157 reported violent events were attributed to delayed treatment. In 22 of 42 reports (52.4%), doctors noted that the violence occurred after referring the patient to higher-level healthcare centers (i.e., from the primary to secondary or tertiary level or from the secondary to tertiary level). In analyzing these specific cases, researchers identified a pattern of violence triggered by patients dying on their way to the referred hospital. Upon taking a closer look at these specific reports, we found that it was common for family members to be hesitant to decide whether or not to take their loved ones to the referred hospital. Some doctors expressed their belief that this hesitancy was partly due to the patients’ party assuming that healthcare professionals were avoiding duties and could do more to help. A handful of doctors noted that they were accused of transferring the patient to their own private practice to charge more money, which also contributed to hesitancy in following referral instructions. Another major cause of a referral delay noted in the doctors’ reports was due to logistical obstacles, such as a lack of accessible transportation at night. In almost all cases in which friends or relatives refused to take the patient to a higher-level healthcare facility, doctors were pressured to provide treatment as best they could.

One primary healthcare doctor reported:

*“Our doctors referred a cardiac patient to the district level hospital in the late evening, but the relatives of the patient spent a few hours deciding. The patient had died by that time. After that, they blamed the doctors for the patient’s death and vandalised our emergency department with the help of some locals. Two of our doctors were injured and lots of valuable instruments were damaged. Later, the police came to control the situation”* (Case 137, Male doctor).

According to the doctors’ posts, the remaining incidents of treatment delay occurred as the patients themselves were unsatisfied with the triage and wait times assigned. Doctors in these cases noted they were busy treating other patients when those who had been waiting became agitated.

*“The doctor was providing treatment to a patient at the emergency unit. At the same time, a guardian of a child patient became very aggressive as he was not getting treatment and physically abused the doctor. It caused a fracture on the fifth metacarpal bone on the doctor’s right hand”* (Case 129, Male doctor).

Incidents like the above illustrate how long hospital wait times, brought on by overburdened systems and too few staff, could be a contributing factor to the dissatisfaction that leads to violence committed against hospital staff and doctors.

### Power Practice

“Power practice,” or intimidation through the use of social or political power by patients or their party, was another common theme identified in the analysis of hospital violence reports. Of 41 reports involving some form of power practice, 21 of them (63.4%) involved the patient or patients’ party trying to use their political or local administrative power to get faster treatment over others who were triaged ahead of them. A surprising finding was that extortion accounted for the power practice in six (14.6%) of the violent incidents in this category. Groups of strong youth demanded money from doctors for social events, such as funding sports or cultural programs. When these doctors refused to donate money, they faced aggression, which often resulted in hospital vandalism. Around 9.8% of the reported events (4 of 41) involving power practice occurred when doctors declined to do home visits or inpatient visits during hours they worked on the emergency ward. Several doctors also recalled asking patients’ accompanying guests to vacate the area during the examination to avoid crowding. These requests also contributed to an escalation of aggressive behavior and unsafe environments in the ward. One doctor from a primary healthcare setting recalls how a request to pay a higher amount for a valid “ticket” to reserve an appointment at a hospital led to an altercation:

*“About 30–40 people came and took part in the hospital vandalism after we did not allow them to enter the hospital without buying a ticket. All patients must buy a ticket from the counter before they can visit the doctor. It only cost BDT 3 but they wanted to buy a ticket with BDT 2 instead of BDT 3 and our staff did not allow that. So, they attacked us and looted our outdoor cash register”* (Case 68, Male doctor).

### Death Declaration

In approximately 18% of the reported incidents (28), the patient’s loved ones turned to violence immediately upon hearing the death declaration. Among these events, 53.5% of the patient deaths (15 of 28) occurred due to chronic illnesses, including stroke, cardiovascular disease, and chronic obstructive pulmonary disease. Other reported cases include miscellaneous events, such as deceased patients before reaching the hospital or dying during resuscitation. Once the doctor declared death, the friends or relatives of patients became aggressive, blaming them for the death. As one of the doctors from a tertiary level hospital described:

*“A patient died accidentally due to excessive bleeding. His sons claimed that their father died due to the negligence of the doctors. They beat an intern doctor for the death of their father. Later they were arrested by the police for this incident”* (Case 131, Male doctor).

### Extreme Violence

A total of eight incidents (5%) were reported that were categorized as most extreme, including sexual assault of female doctors and murder. A majority of these incidents (5 of 8) were female doctors reporting physical and sexual assault by patients’ parties. One doctor working in a secondary-level healthcare setting mentioned:

*“One of our female intern doctors was harassed, which was sexual in nature, during her evening follow-up, by one of the patients’ companions. When we objected to the sexual harassment, they became more agitated and started assaulting us physically”* (Case 16, Female doctor).

Three reports (3 of 8; 37.5%) sadly mentioned that doctors were killed. All of these incidents occurred at private hospitals. One of the reports described one such incident:

*“A female part-time doctor was killed by strangulation by the caretaker of a private hospital during her night duty. While she was resting in her office, he knocked on the door and forcefully tried to enter the room. She could not prevent him from entering the room. Once he entered the room, he tried to rape her but failed. Then he killed her as she tried to stop him from raping her. Later the accused was arrested by the local police and after a few hearings, the court sentenced him to death for killing the doctor”* (Case 118, Female doctor).

This unfortunate case highlights the risks that hospital staff face not only from patients and patients’ caretakers but also from their colleagues. The quoted statement above highlights the need for increased security, staffing, and other hospital resources.

### Care-Seeking Behavior

Patients’ care-seeking behavior was identified as another factor contributing to violent incidents, with around 12% (19) of incidents fitting under this theme. Patients most often became violent when they were unable to get appropriate treatments. In these situations, doctors perceived that the patients acted violently due to resource shortages, including poor hospital-related logistics, ambulance service unavailability, or lack of specialist doctors. A tertiary-level hospital doctor explained the dire consequences of not having high-level expert staff:

*“A patient who attempted suicide by drinking poison was admitted at the hospital at dawn and died around 9am. After that, the relatives complained that the patient died as no specialist doctor visited him and the junior doctors were not capable enough to treat him”* (Case 128, Male doctor).

In some instances, patients became violent after doctors referred them to another hospital or facility for specialized care. These incidents mostly occurred at the primary care level, which often has insufficient facilities. A doctor reported:

*“The doctor suggested an injured patient receive a normal x-ray instead of a digital x-ray, which was not available at that facility. After that, the patient’s caretakers argued with the doctor and beat him in front of the other patients. The doctor got several injuries on his face caused by broken glass from a table. The people who beat him were local politicians”* (Case 39, Male doctor).

The case above highlights the need for all facilities to offer a wide array of services. Even those with esteemed positions in society, like local politicians, resort to violence when faced with inadequate healthcare.

## Discussion

The current study explored the context of WPV in healthcare settings, as reported by doctors on social media. This study’s findings identified occupational risks healthcare providers face that have been documented previously (Khan et al., 2010). According to the WHO, between 8 and 38% of healthcare workers face physical violence at their workplaces (Khan et al., 2010; Tian et al., 2020). Physical violence, verbal aggression, and sexual harassment are all common forms of violence against healthcare providers (Sun et al., 2017). This study included all of these forms of violence, and their potential catalysts were explored. This study’s reports of violence escalated to individuals and mobs destroying hospital property and committing assault and murders, similar to another study conducted in Bangladesh (Hasan et al., 2018).

The majority of violence reported in this study occurred at primary healthcare centers, as was also found in China (Cai et al., 2019). The Bangladesh health system is well structured with three levels of healthcare facilities; The first level, primary healthcare (PHC), comprises three tiers of care, offered at the sub-district, Union (collection of a few villages), and village facilities. The second level of healthcare is the District Hospitals. Tertiary hospitals, specialized hospitals, and medical colleges make up the third level (Islam and Biswas, 2014). Previous studies have found that up to 70% of PHC facilities lack six essential medical devices (thermometers, stethoscopes, blood pressure gauges, weighing scales, and torchlights; Fahim et al., 2019). Large patient volumes heighten risks of violence from dissatisfied patients (Khan et al., 2010; Hasan et al., 2018).

Findings suggest that the lack of resources in PHC centers led to patients being referred to secondary hospitals. Delays of treatment due to decision hesitancy or lack of transportation often result in patient death. Barriers included distance needed to travel, rural transportation issues, patient condition, and lack of understanding the gravity of the situation. A study conducted in Iran suggests that inadequate communication between doctors and patients could also contribute to delays in treatment (Najafi et al., 2017). Treatment dissatisfaction was another factor contributing to violence against doctors, similar to findings from India (Kumar and Roy, 2019). Also similar to findings from this study, a national WeChat-based survey conducted in China found a significant relationship between the announcement of a death declaration and violence against healthcare professionals (Tian et al., 2020). A nationwide study in China estimated that over 20% of WPV in healthcare was associated with the patient’s death (Cai et al., 2019).

Research from Pakistan cited treatment delay as the main contributing factor in violence against doctors, accounting for 13.4% of violent incidents (Shaikh et al., 2020). This study found an even higher percentage of WPV associated with delayed treatment (27%). A lack of resources, poor logistical planning, and unavailability of appropriate staff have been identified as contributors to subpar treatment in Bangladesh (Reddy et al., 2019). In Pakistan, lack of medicines or equipment (6.2%), high patient volume (3.7%), overcrowding due to patient friends and family visiting (3.4%), and referral to other healthcare facilities (3.7%) were found to be significant causes of violence (Baig et al., 2018). Similar findings across these global studies evoke the possibility of public health authorities adopting and investing in already evidenced solutions being implemented elsewhere.

Extreme violence, including homicide and sexual assault, is a common WPV in many high-income countries like the United States and China (Mirza et al., 2012; Ambesh, 2016; Phillips, 2016). Unfortunately, our study also included five reports of sexual assault against female doctors and three murders of on-duty doctors. Lack of security measures and limited knowledge of feasible protection measures contribute to these extreme events.

This study indicates that power practice was the main contributor to violence in 26 cases. Patients and their visitors were reported to use their political power to threaten healthcare providers. Local political leaders and police causing violence in hospitals and primary health centers have also been reported in West Bengal and Maharashtra (Lancet, 2010; Baig et al., 2018; Ghosh, 2018). Political protests against government hospitals and healthcare professionals’ smear campaigns for political gain are common in South Asian countries, including Pakistan, Nepal, and Sri Lanka (Khawaja and Irfan, 2011; Mirza et al., 2012; Magar, 2013; Jiao et al., 2015; Yang et al., 2019).

## Strengths and Limitations

As the data for this content analysis were collected from a social media forum of registered doctors in Bangladesh, they may be prone to reporting bias and not present comprehensive accounts of the reported violent events. Additionally, reports of healthcare professionals committing violence against other healthcare workers are likely to be underreported on a forum such as this, so other means of collecting this hidden data should be explored. Patient perspectives should also be considered for future studies looking to understand the dynamics of WPV in healthcare settings. Due to the lack of proper information in the reported events, we could not retrieve the sex and socio-economic status of the people who caused the violence. The authority of the hospital and doctors mentioned that the attackers left the hospital immediately after the act, so their socio-demographic characteristics could not be identified. Another limitation of this study is the generalizability of findings, as only 157 reported cases were included in the analysis. A large-scale study that includes both patient and doctor accounts is needed to capture a fuller extent of healthcare-based workplace violence more accurately.

Despite these limitations, this study is the first of its kind in Bangladesh to explore patterns of violence against doctors in healthcare settings. Data analyzed comprised first-person narratives of WPV experienced over 6 years, a significant portion of time for a study of this nature. This study identified potential risk factors associated with hospital violence against doctors. Policymakers and hospital administrators should utilize these findings to prevent and reduce violence in the healthcare setting in Bangladesh.

## Conclusion

Healthcare workers play an essential role in the welfare of the country. Further research for a needs-based assessment of healthcare workers should be conducted to elucidate barriers, coping mechanisms, and daily stressors that patients, patients’ parties, and doctors face. Governments and local political bodies must also be involved to ensure a safe work environment for all. The following recommendations may be used as a guide to overcome currently identified barriers:

- Governmental security guards should be posted at hospital entrances. Visitor identification should also be checked during both entry and exit.
- Visitors’ names and addresses should be registered and recorded at the hospital and should be cross-checked with photo identification.
- Hospital authorities should assign visitor passes to reduce crowding.
- Every hospital should have an emergency evacuation plan prepared in case of extreme violence.
- Hospital authorities should create and place emergency helplines throughout the hospital, so workers can quickly seek help if they feel unsafe.
- Training sessions on de-escalation tactics should be conducted and frequently repeated for healthcare workers.

## Data Availability Statement

All posts can be found archived in the news feed of the social media described in the methods section, Platform (https://www.platform-med.org/category/news-event/). Other relevant research data and materials may be made available upon reasonable request to the corresponding author.

## Ethics Statement

Ethical review and approval was not required for the study on human participants in accordance with the local legislation and institutional requirements. Written informed consent for participation was not required for this study in accordance with the national legislation and the institutional requirements.

## Author Contributions

SBK, MR, and MKH conceptualized the idea and designed the study. MR and MMH were involved in retrieving and cleaning the data. SBK analyzed data and drafted the manuscript. MKH and JS were involved in the data coding, analysis, interpretation of findings, and manuscript formatting. SK, EC, and KK guided and supported by critically reviewing the manuscript. All authors contributed to the paper and approved the final version of the manuscript.

## Conflict of Interest

The authors declare that the research was conducted in the absence of any commercial or financial relationships that could be construed as a potential conflict of interest.

## Publisher’s Note

All claims expressed in this article are solely those of the authors and do not necessarily represent those of their affiliated organizations, or those of the publisher, the editors and the reviewers. Any product that may be evaluated in this article, or claim that may be made by its manufacturer, is not guaranteed or endorsed by the publisher.

## Abbreviations

WPV: Workplace violence
NIOSH: National institute for occupational safety and health
US: United States
BMA: British medical association
UK: United Kingdom
BLS: Bureau of labor statistics
WHO: World health organization
UH&FPO: Upazila health and family planning officer
COPD: Chronic obstructive pulmonary disease
PHC: Primary healthcare
UHC: Upazila health complex
UHFWC: Union health and family welfare center
CC: Community clinic
